# Bacterial Communities of Surface Mixed Layer in the Pacific Sector of the Western Arctic Ocean during Sea-Ice Melting

**DOI:** 10.1371/journal.pone.0086887

**Published:** 2014-01-31

**Authors:** Dukki Han, Ilnam Kang, Ho Kyung Ha, Hyun Cheol Kim, Ok-Sun Kim, Bang Yong Lee, Jang-Cheon Cho, Hor-Gil Hur, Yoo Kyung Lee

**Affiliations:** 1 Korea Polar Research Institute, KIOST, Incheon, Republic of Korea; 2 School of Environmental Science and Engineering, Gwangju Institute of Science and Technology, Gwangju, Republic of Korea; 3 Division of Biology and Ocean Sciences, Inha University, Incheon, Republic of Korea; Stazione Zoologica, Italy

## Abstract

From July to August 2010, the IBRV *ARAON* journeyed to the Pacific sector of the Arctic Ocean to monitor bacterial variation in Arctic summer surface-waters, and temperature, salinity, fluorescence, and nutrient concentrations were determined during the ice-melting season. Among the measured physicochemical parameters, we observed a strong negative correlation between temperature and salinity, and consequently hypothesized that the melting ice decreased water salinity. The bacterial community compositions of 15 samples, includicng seawater, sea-ice, and melting pond water, were determined using a pyrosequencing approach and were categorized into three habitats: (1) surface seawater, (2) ice core, and (3) melting pond. Analysis of these samples indicated the presence of local bacterial communities; a deduction that was further corroborated by the discovery of seawater- and ice-specific bacterial phylotypes. In all samples, the *Alphaproteobacteria*, *Flavobacteria,* and *Gammaproteobacteria* taxa composed the majority of the bacterial communities. Among these, *Alphaproteobacteria* was the most abundant and present in all samples, and its variation differed among the habitats studied. Linear regression analysis suggested that changes in salinity could affect the relative proportion of *Alphaproteobacteria* in the surface water. In addition, the species-sorting model was applied to evaluate the population dynamics and environmental heterogeneity in the bacterial communities of surface mixed layer in the Arctic Ocean during sea-ice melting.

## Introduction

During summer, water from the north Pacific enters the Arctic Ocean after passing through the Bering Strait, intermixing with freshwaters from melting ice. The surface mixed layer is strongly affected by wind, producing a distinctive water mass of density-defined layers in the upper water column. Locally-formed water masses are identified by their temperature and salinity, where distinctive microbial communities can persist [1]–[3]. Microbial communities in the surface waters would be affected by environmental changes resulting from melting sea-ice because the amount of sea-ice is dramatically reduced in this area during summer. Understanding the structure of the microbial community and the interaction between coexisting populations and environmental factors may elucidate how microbial communities respond to environmental changes.

The metacommunity concept in community ecology is helpful in understanding the distribution and interaction of local communities [4]. The species-sorting model of the metacommunity concept describes variation in the abundance and composition of bacterial communities. It suggests that individual species respond to environmental heterogeneity, and, therefore, certain local conditions may favor some species and not others. If the species-sorting process is effective, different bacterial communities should be found in different habitats. Moreover, at least some of the taxa in bacterial communities should resemble those in a similar habitat. On the other hand, species-sorting effects on bacterial community composition can be relatively weak when other metacommunity aspects, such as the source-sink dynamics and neutral model, operate at the same time. That is, the species-sorting process may be influenced by other elements during the formation of a bacterial community, or it may depend on how many generalist (commonly occurring in the surrounding habitat regardless of environmental conditions) and specialist (habitat-specific) taxa are present in a community [5].

In the species-sorting model, selection by local environmental conditions plays a key role in the composition of bacterial communities. The species-sorting model has been applied to bacterial communities in many environmental conditions [6]–[12]. To our knowledge, however, there is no case study using the species-sorting model to assess the marine bacterial community in Arctic surface water during the sea-ice melting season. We assume that the species-sorting model is a specific process, useful for understanding changes in bacterial community patterns caused by the introduction of fresh water during sea-ice melting in the north Pacific.

The aims of this study were to examine the composition of the bacterial community in Arctic marine environments and to understand the population dynamics of bacterial communities in surface water mixing with fresh water. A pyrosequencing approach targeting the V1–V3 region of the 16S rRNA gene was used to survey the bacterial communities in the Arctic Ocean. The physicochemical parameters temperature, salinity, fluorescence, and nutrient concentrations were measured to estimate differences in habitat difference and determine the effect of environmental factors.

## Materials and Methods

### Sample Collection and Analysis of Environmental Factors

**Figure 1:**
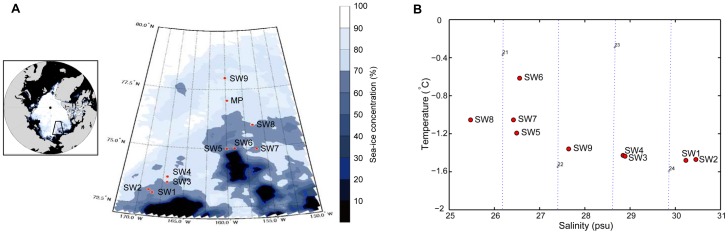
Sampling site descriptions. The (A) sea-ice monitoring in the Pacific sector and (B) T-S diagram are indicated. Seawater samples are denoted SW1 to SW9, and MP is the sea-ice station for melting pond and ice core samples. Sea-ice concentrations around the Pacific sector of the Arctic Ocean were collected from the AMSR-E database during the expedition. Temperature and salinity data were collected from experimental sites at a 7-m depth, using physical sensors equipped in the CTD rosette system.

The first expedition of the IBRV *ARAON* to the Pacific sector of the Arctic Ocean proceeded from July to August in 2010. Nine stations were selected for surface water sampling at a depth of 7 m (Fig. 1A; SW1 to SW9). Samples for melting ponds and ice-core were collected from regions near the sea-ice station (Fig. 1A; MP). Surface water samples (SW1 to SW9) were collected using a CTD rosette system (SeaBird, SBE-911plus) and immediately passed through 3-µm pore membrane filters (ADVANTEC, Japan) to separate suspended particles and eukaryotes. Finally, filtration with a 0.2-µm pore membrane (ADVANTEC) was performed to capture bacteria. Water samples in four melting ponds were collected using a Niskin sampler and were processed in the same manner as the seawater samples. The sea-ice core (103 cm) was cut into two parts, the ice-top (IT: 41 cm) and the ice-bottom (IB: 61 cm). Both of the ice core parts were melted separately, after a sterile knife was used to peel a thin layer from the surface of the ice core for decontamination, and then filtered according to their respective protocol as described above. The 0.2-µm pore-sized membranes were stored in a deep-freezer (−80°C) in the *ARAON* and transported, on ice, back to the laboratory in KOPRI for DNA extraction. No specific permits were required for this study because our sampling stations are outside of any EEZ in the Arctic Ocean (73°01′ 78°00′ N, 156°14′ 168°56′ W, see Table S1), and this field study did not involve endangered or protected species.

Temperature, salinity, and fluorescence were characterized for all seawater samples by sensors in the CTD rosette system, and a nutrient analysis was conducted to estimate the concentrations of PO_4_^3−^, NO_2_^−^+NO_3_^−^, NH_4_^+^, and SiO_2_. To examine the status of sea-ice formation around the research area, data for the concentrations of sea-ice on the water’s surface were collected from the dataset measured by the AMSR_E sensor, which is equipped in the satellite Aqua, and converted by a Matlab program.

### DNA Extraction, Amplification, and Sequencing of the 16S rRNA Gene

Total DNA was extracted according to a conventional phenol/chloroform DNA isolation method (http://openwetware.org/wiki/Phenol/chloroform_extraction) and purified using a commercial kit (Qiagen, Hilden, Germany). Bacterial 16S rRNA gene sequences were amplified from the extracted gDNA using primers targeting the V1 to V3 regions. Primers were V1–21F (5′-*CCTATCCCCTGTGTGCCTTGGCAGTC*-*TCAG*-AC-GAGTTTGATCMTGGCTCAG-3′) and V3-537R (5′ *CCTATCCCCTGTGTGCCTTGGCAGTC*-*TCAG*-[*X*]-AC-WTTACCGCGGCTGCTGG-3′). The adaptor, key sequences, and a common linker AC are indicated in italics, whereas 16S rRNA gene-specific sequences are underlined. Symbol [*X*] denotes a 7–9 nucleotide-long barcode uniquely designed for each sample. PCR reactions were performed using DreamTaq^Tm^ Green PCR Master Mix (Fermentas, EU) and the following conditions: initial denaturation at 94°C for 5 min, followed by 30 cycles of denaturation at 94°C for 30 s, annealing at 55°C for 30 s, elongation at 72°C for 1 min 20 s, and a final extension at 72°C for 10 min. PCR amplicons were purified using the Qiaquick PCR purification kit (Qiagen), and concentrations were measured by spectrometry (NanoDrop Technologies, Wilmington, MA). The purified amplicons from samples were mixed in equimolar amounts and subjected to pyrosequencing. Pyrosequencing was performed by Macrogen Incorporation (Seoul, Korea) using a 454 GS FLX Titanium Sequencing System (Roche). All sequences can be accessed from the Sequence Read Archive at EMBL database, under accession number ERP002081.

### Analysis of Pyrosequencing Results

Analysis of pyrosequencing data was performed using the MOTHUR program Ver. 1.23 [13], according to the protocol described by Schloss et al. (http://www.mothur.org/wiki/Schloss_SOP) [14]. In brief, sequencing reads from the different samples were separated by unique barcodes. Then, the barcode, linker, and PCR primer sequences were removed from the original sequencing reads. The resultant sequencing reads were subjected to a filtering process, which included quality trimming and chimera removal. After aligning the sequencing reads against the pre-aligned SILVA reference database, quality-filtered sequencing reads were used to calculate a distance matrix. Operational taxonomy units (OTUs) were clustered by the furthest neighbor algorithm, with similarity cutoffs of 99%, 97%, 95%, and 90%, the results of which were then used to compare bacterial communities of samples. In the alpha diversity analysis, species richness was estimated by the ACE and Chao1 indices, whereas species evenness was calculated by Shannon and Simpson indices [15]. To analyze beta diversity, the principal coordinates analysis (PCoA) was used at a 99% similarity cutoff.

### Taxonomic and Phylogenetic Analyses

Taxonomic classification of the individual reads was achieved using the NAST algorithm in the Greengenes database (http://greengenes.lbl.gov) [16]. Detailed phylogenetic analyses were performed for representative sequences retrieved from 26 major OTUs, with a frequency over 1% in each sample, after OTU clustering with a cutoff level of 99%. Retrieved sequences were aligned by the SINA online aligner, imported into the ARB database (SSURef_108), and used to determine phylogenetic affiliation. To construct a phylogenetic tree, aligned sequences were exported into MEGA 5.05 along with other related sequences [17]. A phylogenetic tree was constructed using the neighbor-joining method with 100 bootstrap tests. Additionally, BlastN analyses were performed against a non-redundant GenBank database and, when needed, against the ARB database for more accurate phylogenetic assignments.

### Statistical Analyses

Prior to statistical analyses, the sequences were normalized in the following way: the relative abundance of each phylotype was calculated, and minor populations with a lower relative abundance (less than 1% in the sample) were excluded from further analyses. To verify the link between physicochemical parameters and major phylotypes among the samples, a nonparametric correlation analysis was performed using the Spearman’s rho test. A further linear regression analysis was performed to infer tendencies toward the significant relation suggested by the Spearman test. Significantly correlated parameters were included in the linear regression analysis, and the data were checked for normality (normal p-p plot), auto-regression (Durbin-Watson), and outliers (standardized residual) from regression diagnostics. All statistical analyses were performed by the SPSS program ver. 16.0 (SPSS Institute, Cary, NC).

## Results

### Sea-ice Distribution and Seawater Properties

Various samples were collected from the Pacific sector of the Arctic Ocean: seawater (n = 9) from the surface mixed layer, brine water (n = 4) from melting ponds, and a sea-ice core that was further divided into upper and lower sections (n = 2). Pyrosequencing analysis was used to determine the bacterial community structure in these samples. The concentrations of sea-ice on the surface around the sampling stations in the Pacific sector were projected into the image shown in Figure 1A. The area from SW1 to SW7 was mostly covered by ice floes, and sea-ice concentrations were over 50%. In contrast, the SW8 to SW9 area, including the sea-ice station (MP), was occupied by a sheet of sea-ice more than 2 m thick, and had a sea-ice concentration over 70%.

The distribution of water mass in the seawater sampling stations is shown in the T-S diagram (Fig. 1B). Samples were classified into five groups: (1) SW1 and SW2, (2) SW3 and SW4, (3) SW5 to SW7, (4) SW8, and (5) SW9 by water mass characteristics. Interestingly, a remarkable temperature change (−0.6 to −1.2°C) was measured in the SW5 to SW7 group, suggesting that there was a subtle change in the water mass as a consequence of Arctic sea-ice reduction, rather than a measurement bias. The salinity of the samples remarkably decreased from SW1 to SW9, and this pattern significantly correlated with temperature (p<0.01, r = −0.867; Table 1).

**Table 1:** Statistical correlation analysis of seawater to determine an environmental link.

	Temperature	Salinity	Fluorescence	PO_4_^3−^	NO_2_^−^+NO_3_^−^	NH_4_^+^	SiO_2_
Temperature							
Salinity	−**0.867/0.002** *						
Fluorescence	0.600/0.088	−0.383/0.308					
PO_4_^3−^	0.111/0.777	−0.06/0.879	−0.145/0.711				
NO_2_^−^+NO_3_^−^	0.390/0.300	−0.225/0.56	−0.104/0.79	0.429/0.249			
NH_4_^+^	0.577/0.104	−0.644/0.061	0.301/0.431	−0.103/0.793	0.444/0.232		
SiO_2_	−2.582887701	0.367/0.332	0.150/0.700	−0.443/0.233	−0.303/0.428	0.192/0.620	

Physicochemical parameter values for seawater (described in the Table. S1) were analyzed by the Spearman’s rho test in SPSS. Bold number is significant at p<0.05.

* Correlation Coefficient/p-value.

The ice core and the closed melting pond were distinctively different from the seawater samples in the salinity range. Two types of melting ponds were found: open and closed. The salinity of open melting ponds (MP1 to MP3) was all measured to approximately 26.0 psu, indicating that these melting pond waters were mixed with seawater when there was a hole in the bottom of the ponds. In contrast, the only closed melting pond sampled, MP4, showed much lower salinity (13.0 psu; Table 1) because the bottom of the closed pond was not connected to seawater (Fig. S1). The ice core sample was determined to have the lowest salinity among all the samples, with the bottom of the ice core (IB; 2.4 psu) being slightly more saline than the top (IT; 1.6 psu).

Location and physicochemical parameters, such as temperature, salinity, fluorescence, and nutrient concentrations (PO_4_^3−^, NO_2_^−^+NO_3_^−^, NH_4_^+^ and SiO_2_), for seawater samples (SW1 to SW9) are shown in Table S1. Among the parameters assayed, only salinity and temperature significantly correlated with each other (Table 1). Generally, oceanic nutrients and organic matter spread rapidly and widely in surface water and are distinguishable below the surface layer in which the water is turbulently mixed by wind. For instance, the concentrations of most nutrients were retained at a certain range in seawater (SW1 to SW9). Nevertheless, concentrations of SiO_2_ in samples SW1 and SW2 were relatively higher than in the others (Fig. S2D). This finding indicates that there is a distinction between melt-affected seawaters and Pacific waters that have flowed in from outside of the Arctic Ocean.

### Bacterial Diversity and Taxonomic Classification

**Figure 2:**
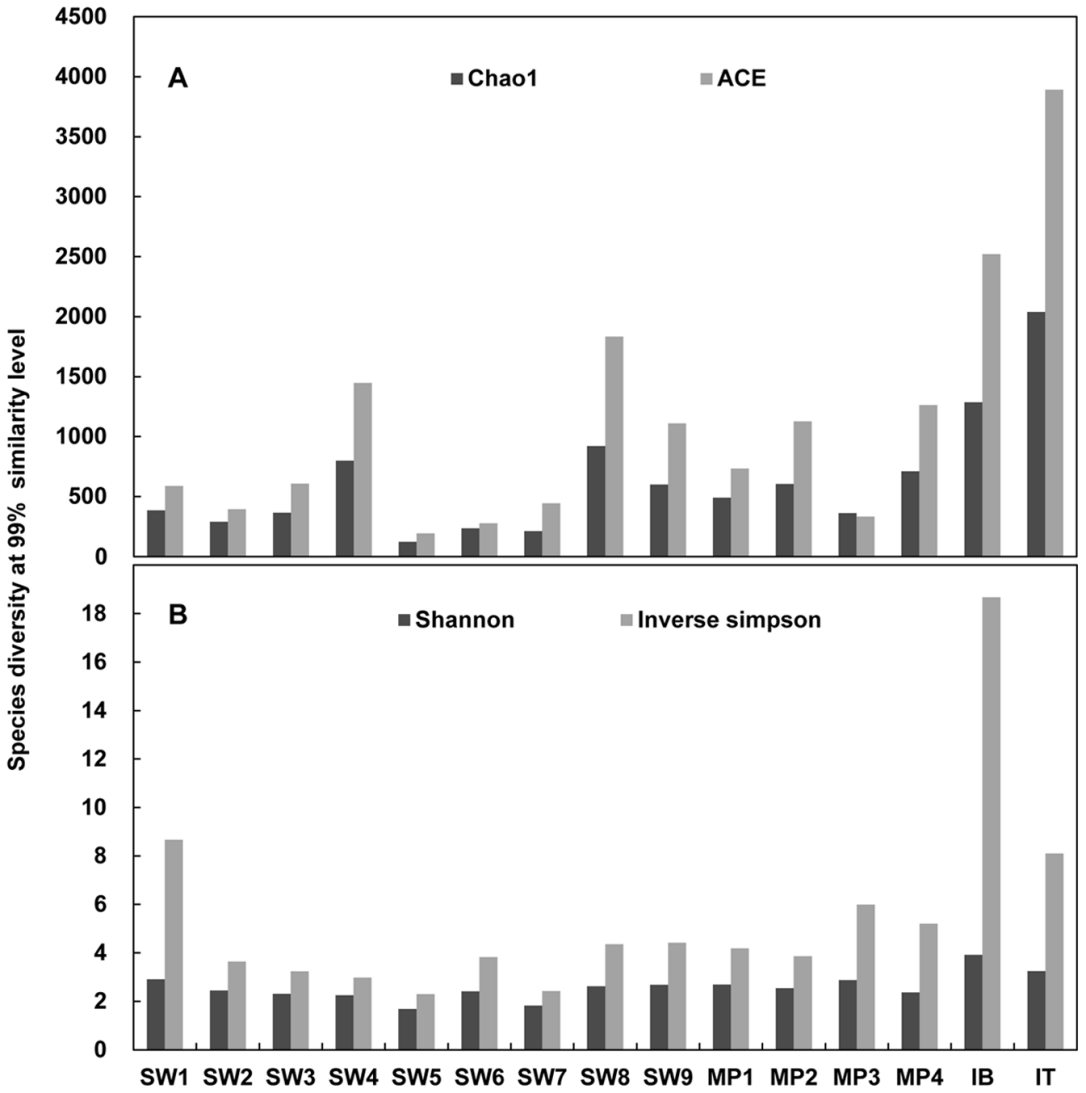
Alpha diversity analysis. Species diversity is represented on a local scale (for all samples) at a 99% similarity level (patterns of 97%, 95%, and 90% are similar with 99%, not shown here). (A) Species richness is expressed by the Cho1 and ACE indices and denotes the number of taxa in a community. (B) Species evenness denotes the measure of the relative abundance of taxa and is expressed by the Shannon and Simpson indices. The values of the Simpson index were converted to be shown in the same graph and are represented by the Inverse Simpson Index.

**Figure 3:**
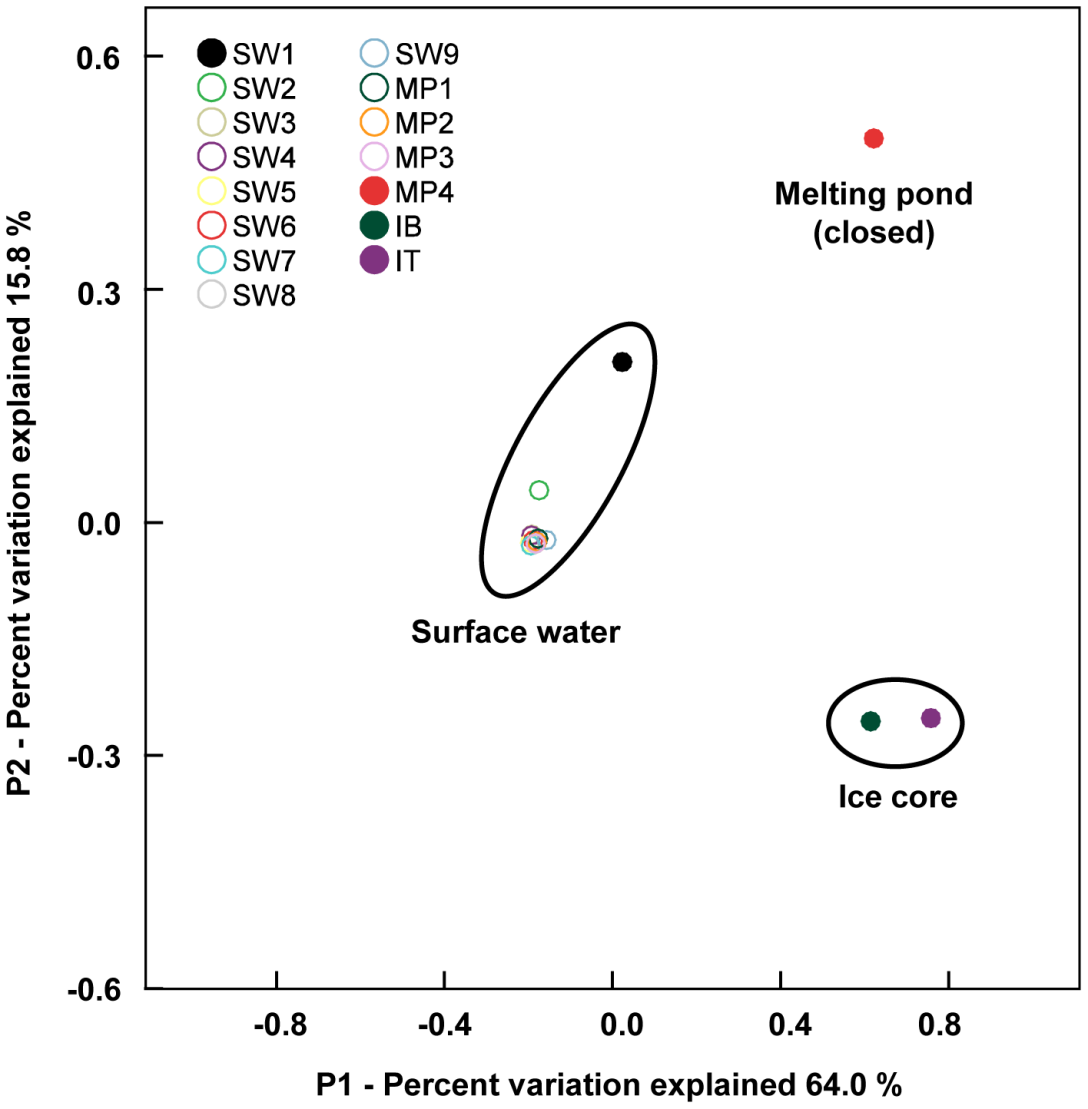
Beta diversity analysis. Species diversity of bacterial communities is expressed by the Principal Coordinates Analysis (PCoA) and represents the differentiation among samples. Comparison of the variation between samples is explained along the P1 (64.0%) and P2 (15.8%) axes. SW1 to SW9 represent seawater samples (described in Fig. 1A). MP1 to MP4 represent water samples collected from melting ponds, and IB and IT represent the bottom and top layers of the ice core, respectively, in the sea-ice station (described in Fig. S1). Ellipses were drawn manually to aid in visualizing results.

A total of 64,070 sequencing reads were obtained from the 15 samples after quality trimming and chimera removal. In analyses of alpha diversity (Fig. 2), species richness was higher in the ice core (IB and IT) than in seawater (SW1 to SW9) and melting pond water (MP1 to MP4) at all similarity thresholds for OTU clustering (99%, 97%, 95%, and 90%). Species evenness was also higher in the ice core. Interestingly, the alpha diversity indices seem to indicate an irregular pattern for the SW5 to SW7 samples among seawaters, as shown in the T-S diagram (Fig. 1B). In the beta diversity analysis using PCoA (Fig. 3), the bacterial communities were clustered into three major groups: surface water, melting pond (closed), and ice core. All seawater and melting pond samples formed a cluster, with SW1 and SW2 comprising slightly different communities as compared to other samples. In contrast to open melting ponds, the closed melting pond sample (MP4) occupied a distinct position. The ice core (IB and IT) also formed a separate cluster.

**Figure 4:**
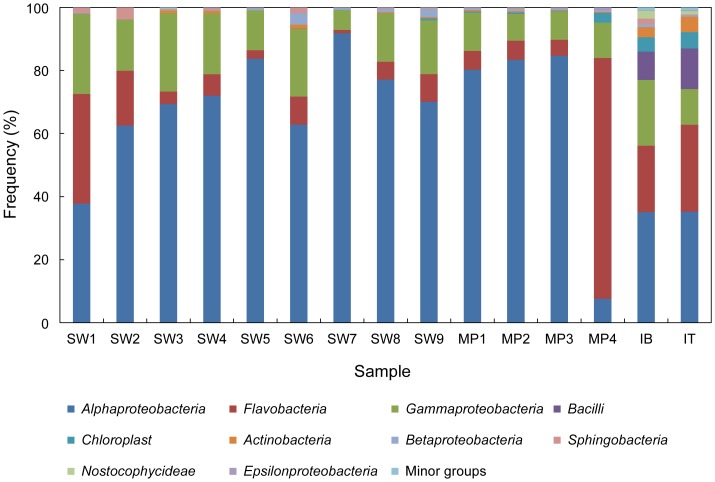
Bacterial community in class level. The minor groups denote the sum of rare phylotypes that were arbitrarily defined as having a frequency of <1% within a sample. The percentage of all taxa is described in Table S2.

To further characterize the differences in the bacterial communities among the samples, taxonomic assignments of all sequencing reads were performed by comparison with the Greengenes database at the class level (Fig. 4). While *Alphaproteobacteria* (62.8%), *Flavobacteria* (15.8%), and *Gammaproteobacteria* (15.6%) were the major taxonomic groups in all samples, the proportions of these taxa varied among all the samples. The proportion of *Alphaproteobacteria* exceeded 50% in all seawater (except SW1) and open melting pond samples, which together formed a major cluster in the PCoA plot (Fig. 3). The SW1 sample, which was least closely located to the surface water in the PCoA plot among seawater samples, had a relatively lower proportion of *Alphaproteobacteria* but a higher proportion of *Flavobacteria* when compared with other seawater samples. The proportion of *Alphaproteobacteria* was relatively lower in the ice core sample. The ice core sample (IB and IT) contained elevated levels of *Actinobacteria* and *Bacilli*, both of which were present in very low levels in other samples. On the other hand, the closed melting pond (MP4) had the highest level of *Flavobacteria*, with a significantly reduced *Alphaproteobacteria* population (Fig. 4).

In a rank correlation test of the major taxonomic groups, the frequency of *Alphaproteobacteria* presence in the surface water was inversely related to that of *Flavobacteria* (r = −0.9, p<0.01) and *Gammaproteobacteria* (r = −0.8, p<0.05) (Table 2). To assess the phylogenetic composition of bacterial assemblages at finer levels, a detailed analysis was performed for representative sequencing reads, each of which was retrieved from major OTUs clustered at a similarity level of 99%. We selected 26 OTUs out of the total 3,182, which contributed more than 1% of the population frequency in at least one sample. Representative sequences from the 26 major OTUs could be grouped into 19 phylogenetic clades at a genus or higher taxonomic level (Fig. S3). Among the 19 phylogenetic clades, there were 6 clades of *Alphaproteobacteria*, 7 of *Gammaproteobacteria*, 3 of *Flavobacteria*, and 1 clade each from *Bacilli* and *Actinobacteria*. The phylogenetic position of the remaining clade (ARC14) could not be resolved because of low similarity to any known bacterial lineage.

**Table 2:** Correlation between major phylotypes in samples.

	Correlation Coefficient/p-value		
	*Alphaproteobacteria*	*Flavobacteria*	*Gammaproteobacteria*
*Alphaproteobacteria*			
*Flavobacteria*	−**0.889/p<0.01**		
*Gammaproteobacteria*	−0.496/p>0.05	0.304/p>0.05	

*Alphaproteobacteria*, *Flavobacteria,* and *Gammaproteobacteria*, the most predominant phylotypes in all total 15 samples (shown in Fig. 4.), were analyzed by nonparametric correlation (Spearman’s rho test). The bold number is significant at p<0.01.

Most of the 19 major phylogenetic clades demonstrated habitat preferences when their distribution in the samples was analyzed (Table 3): some clades were predominantly found in seawater and open melting pond samples, whereas others were preferentially detected in the closed melting pond and ice core samples. All *Alphaproteobacteria* clades, except *Octadecabacter*, were relatively ubiquitous and predominant in seawater-related samples, including the open melting ponds (Table 3). On the other hand, *Flavobacteria* clades were not ubiquitous in the seawater-related samples; most of them were readily found in the ice-related samples (both closed melting pond and ice core). *Actinobacteria* and *Bacilli* occurred primarily in the ice core.

**Table 3:** Distribution of the 26 major OTUs.

	Percentage (%) of major OTUs in the sampling sites															
OTUs	SW1	SW2	SW3	SW4	SW5	SW6	SW7	SW8	SW9	MP1	MP2	MP3	MP4	IB	IT	Phylotypes
	(30.2)	(30.5)	(28.9)	(28.8)	(26.5)	(26.6)	(26.4)	(25.5)	(27.6)	(26.0)	(26.0)	(26.0)	(13.0)	(2.4)	(1.6)	
ARC1	24.3	50.9	54.4	57.0	65.0	49.4	63.0	45.4	36.8	45.1	46.8	48.7	3.4	7.8	1.9	SAR11 group I (AP)
ARC2	4.5	3.4	5.9	0.0	8.5	0.0	10.4	12.0	15.3	12.7	11.3	12.2	0.8	3.5	0.7	RCA clade (AP)
ARC3	0.0	0.0	4.9	4.1	0.0	7.6	1.7	3.9	0.0	1.9	2.6	1.9	0.0	0.4	0.0	SAR86 clade (GP)
ARC4	0.2	0.3	1.2	1.0	2.1	4.3	3.6	4.4	2.6	2.7	3.4	3.3	0.0	0.0	0.0	SAR11 group III (AP)
ARC5	0.3	0.1	2.9	3.2	4.2	7.4	2.1	2.7	2.0	3.1	2.0	1.9	0.0	0.4	0.1	SAR86 clade (GP)
ARC6	18.3	6.0	0.0	0.0	0.0	0.0	0.0	0.0	0.0	0.9	0.0	0.0	0.0	0.0	0.0	*Polaribacter* (FB)
ARC7	3.3	2.0	3.9	4.9	2.3	2.0	0.0	1.2	3.0	0.6	0.5	0.4	0.0	0.0	0.0	Arctic96BD-19 clade (GP)
ARC8	0.3	0.5	0.4	0.7	0.2	0.8	3.8	1.0	3.3	3.5	4.3	4.3	0.0	1.2	0.3	SAR11 group II (AP)
ARC9	10.5	7.8	0.0	0.0	0.0	0.0	0.0	1.1	0.0	2.4	0.0	0.0	0.0	0.0	0.0	*Balneatrix* (GP)
ARC10	1.3	1.0	5.2	1.3	0.0	1.0	0.4	1.5	0.0	1.7	0.6	1.8	0.0	0.0	0.0	SAR86 clade (GP)
ARC11	2.1	2.3	0.1	0.4	1.3	3.7	0.0	1.8	0.9	0.7	0.7	0.3	0.0	0.0	0.0	NS5 marine group (FB)
ARC12	0.0	0.0	1.7	2.3	0.7	3.4	0.6	0.8	0.7	1.2	0.9	1.0	0.0	0.2	0.0	SAR11 clade, Surface 4 (AP)
ARC13	0.5	0.0	3.5	3.3	0.0	0.6	0.1	1.5	0.0	0.0	0.1	0.5	0.0	0.0	0.0	SAR92 clade (GP)
ARC14	0.9	0.1	0.0	0.6	2.6	0.2	1.7	4.8	2.4	4.6	4.3	4.3	0.0	0.9	0.2	unknown
ARC15, ARC23	0.0	0.0	0.0	0.0	0.0	0.0	0.0	0.0	0.0	0.0	0.0	0.0	1.0	14.5	28.4	*Octadecabacter* (AP)
ARC16	0.0	0.0	0.0	0.0	0.0	0.0	0.0	0.0	0.0	0.0	0.0	0.0	0.3	4.7	4.5	*Candidatus*Endobugula(GP)
ARC17, ARC26	0.0	0.0	0.0	0.0	0.0	0.0	0.0	0.0	0.0	0.0	0.0	0.0	0.0	7.9	11.8	*Bacillus* (GPB)
ARC18	0.0	0.2	0.0	0.0	0.0	0.0	0.0	0.0	0.0	0.1	0.0	0.0	10.4	0.0	0.0	*Polaribacter* (FB)
ARC19	0.0	0.0	0.2	0.0	0.0	0.0	0.0	0.0	0.1	0.0	0.2	0.0	0.0	2.6	4.1	*Actinobacteria* (GPB)
ARC20, ARC24	0.0	0.0	0.0	0.0	0.0	0.0	0.0	0.0	0.0	0.0	0.0	0.0	31.5	0.7	3.4	*Polaribacter* (FB)
ARC21	0.0	0.0	0.0	0.0	0.0	0.0	0.0	0.0	0.0	0.0	0.0	0.0	0.0	5.6	0.0	BD7-8 Marine group (GP)
ARC22	0.0	0.0	0.0	0.0	0.0	0.0	0.0	0.0	0.3	0.0	0.2	0.2	26.5	11.2	17.0	*Persicivirga* (FB)
ARC25	0.0	0.0	0.0	0.0	0.0	0.0	0.0	0.0	0.0	0.0	0.0	0.0	9.9	4.1	4.8	*Glaciecola* (GP)
Minor groups	33.5	25.4	15.7	21.2	13.1	19.6	12.6	17.9	32.6	18.8	22.1	19.2	16.2	34.3	22.8	

The 26 OTUs accounted for more than 1% in each sample, and the minor groups denote the sum of rare OTUs that were arbitrarily defined as having a frequency of <1% within a sample. Numbers in parentheses indicate the salinity of each sample. Abbreviations in parentheses: GP, *Gammaproteobacteria*; AP, *Alphaproteobacteria*; FB, *Flavobacteria*; GPB, Gram-positive bacteria.

Even though the distribution of the major phylogenetic clades broadly depended on habitat type, there were subtle differences among clades and samples. SAR11 group I (ARC1), a major bacterial lineage found in seawater, was also the most predominant group in the Pacific sector of the Arctic Ocean. The SAR11 group I lineage was more dominant in the seawater-related samples than in the ice-related samples. Other SAR11 lineages, such as SAR11 group II (ARC8), group III (ARC4), and surface 4 clade (ARC12), were also ubiquitous in the seawater-related samples, even though they were not present in as high a proportion as SAR11 group I (ARC1). The RCA clade of *Alphaproteobacteria* (ARC2) was the second most dominant in the seawater-related samples, except for samples SW4 and SW6, and was as similarly ubiquitous as the SAR 11 clades. Thus, the distribution pattern of *Alphaproteobacteria* is primarily explained by SAR11 group I and RCA clade abundance. In contrast to *Alphaproteobacteria*, the distribution of *Flavobacteria* in seawater-related samples was not fully explained by the abundance of its major clades. A *Polaribacter* clade, represented by ARC18, ARC20, and ARC24, was almost exclusively found in the ice-related samples at very high abundance. Another *Polaribacter* clade (ARC6) mainly occurred in samples SW1 and SW2, where the influence of the Pacific water may be a factor. Among the *Flavobacteria* clades affiliated with the major OTUs, the NS5 marine group (ARC11) was less abundant but nearly ubiquitous in the seawater-related samples.

The overall distribution patterns of the other phylogenetic clades also differed largely between seawater- and ice-related samples, as implicated by the beta diversity analysis. Even though the distribution of the identified phylotypes broadly depended on the two types of habitats, there was a subtle difference within each habitat. For example, four clades, including Arctic96BD-19 (ARC7), SAR92 (ARC13), SAR86 (ARC3, ARC5, and ARC10), and the unknown clade (ARC14), were similarly distributed in seawater-related samples, but the *Balneatrix* clade (ARC9) was abundant in SW1 and SW2.

Some clades were most prevalent in the closed melting pond, followed by the ice core, with almost complete absence in seawater-related samples. The *Octadecabacter* clade (ARC15 and ARC23) and the *Candidatus* Endobugula clade (ARC16) were the most abundant in the ice core, but were absent in seawater-related samples and the closed melting pond. This pattern also appeared for the gram-positive bacteria *Bacillus* (ARC17 and ARC26) and *Actinobacteria* (ARC19). Distribution patterns were similar between the *Persicivirga* clade (ARC22) and the *Glaciecola* clade (ARC25). The BD7-8 marine group (ARC21), however, was present only in the bottom of ice core (IB).

### Correlation Analysis between Major Phylotypes (Class Level) and Physicochemical Parameters

**Figure 5:**
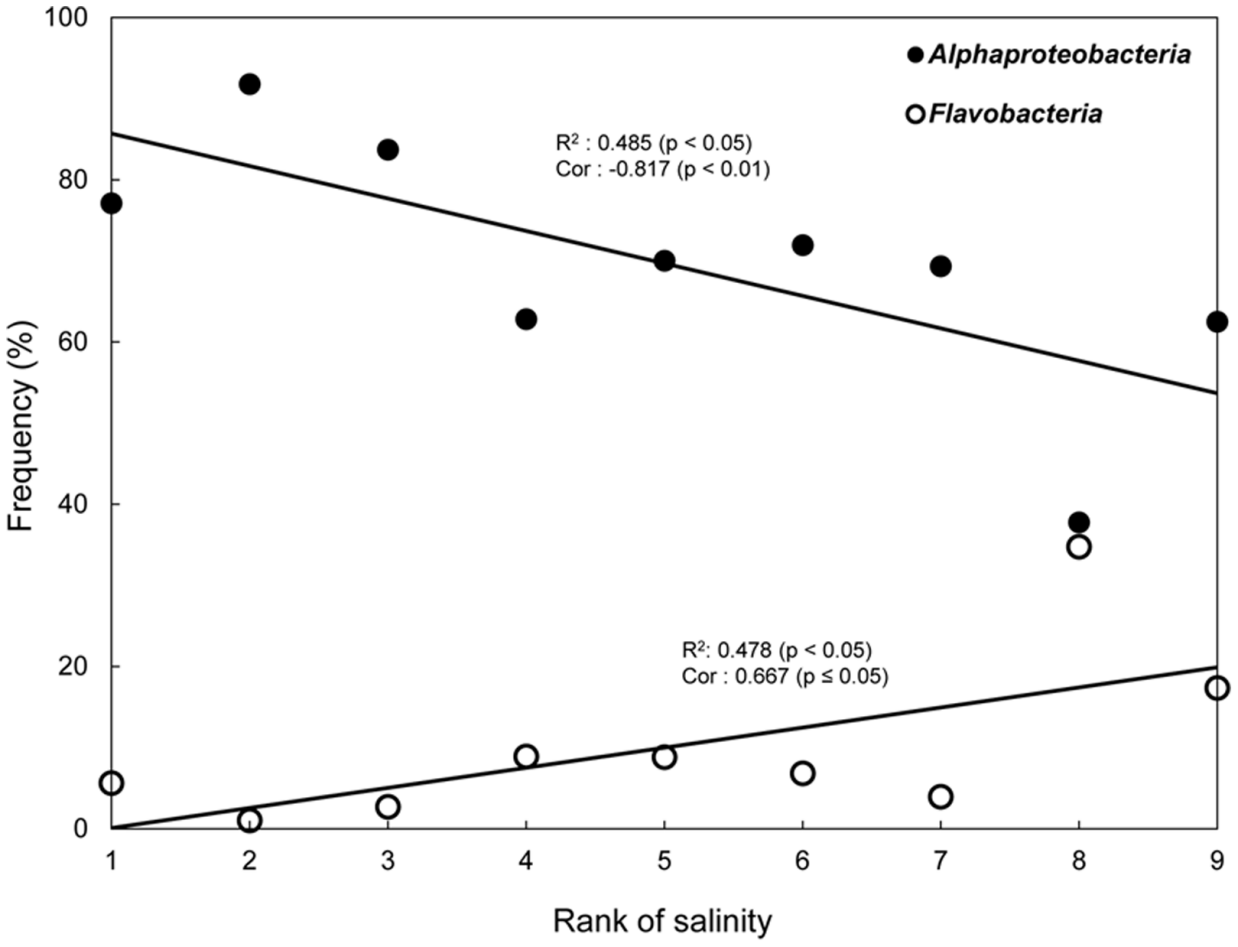
Abundance of *Alphaproteobacteria* and *Flavobacteria* in association with salinity change. The correlation coefficients (Cor) with the associated p-values (described in Table 4) are shown for *Alphaproteobacteria* and *Flavobacteria*. The regression coefficient (R^2^) was calculated by linear regression analysis. The X-axis scale (rank of salinity) assigns 1 to the smallest rank of salinity. Filled circles = frequency of *Alphaproteobacteria* along the salinity change; open circles = frequency of *Flavobacteria* along the salinity change.

The nonparametric correlation analysis between major groups (*Alphaproteobacteria*, *Flavobacteria*, or *Gammaproteobacteria*) and physicochemical parameters was performed with seawater samples. A significant correlation was observed with salinity (Table 4); salinity was correlated with *Alphaproteobacteria* (r = −0.817, p<0.01) and *Flavobacteria* (r = 0.667, p<0.05) (Fig. 5). This result is consistent with an inverse correlation in the abundance of *Alphaproteobacteria* and *Flavobacteria* (Table 2). Causality for the correlation between salinity and these phylotypes was determined by linear regression analysis (Fig. S4). The abundance of *Alphaproteobacteria* was explained by salinity at an accuracy of 48.3% (p<0.05), but both SAR11 group I and the RCA clade, the most abundant and ubiquitous phylogenetic clusters of *Alphaproteobacteria*, showed no statistical significance. Similarly, the abundance of *Flavobacteria* was also explained by salinity (47.8%, p<0.05), and none of the *Flavobacteria* clade showed a statistically significant pattern. However, most of the *Flavobacteria* clades were neither ubiquitous nor dominant in seawaters, which can result in a statistical bias. *Gammaproteobacteria* was not correlated with any physicochemical parameters.

**Table 4:** Nonparametric correlation (Spearman’s rho) test between major phylotype and physicochemical parameters.

Major phylotypes	Correlation Coefficient (p-value) with each physicochemical parameter						
	Temperature	Salinity	Fluorescence	PO_4_^3−^	NO_2_^−^+NO_3_^−^	NH_4_^+^	SiO_2_
*Alphaproteobacteria*	0.617	−**0.817**	0.333	0.468	0.121	0.410	−0.283
	(0.077)	**(0.007)**	(0.381)	(0.204)	(0.756)	(0.273)	(0.460)
*Flavobacteria*	−0.500	**0.667**	−0.317	−0.451	0.130	−0.126	0.417
	(0.170)	**(0.050)**	(0.406)	(0.223)	(0.739)	(0.748)	(0.265)
*Gammaproteobacteria*	−0.517	0.650	−0.567	−0.196	0.104	−0.243	−0.017
	(0.154)	(0.058)	(0.112)	(0.614)	(0.790)	(0.529)	(0.966)

In this test, only seawater samples (SW1 to SW9) were analyzed except for melting pond (MP1 to MP4) and ice core (IB and IT) samples. Bold numbers are significant at p ≤ 0.05.

## Discussion

The water mass characteristics and physicochemical parameters of collected samples reflect environmental changes in the surface mixed layer of the Arctic Ocean during summer. Summer water originating in the Pacific Ocean and entering through the Bering Strait could meet relatively low-salinity water produced by sea-ice melting and freshwater input from the surrounding continents. Temperature and salinity in Arctic seawater were generally associated with the sea-ice concentration despite the intra-variation between SW5 to SW7, shown in the T-S diagram, suggesting that this area is affected by locally-formed water masses from the Beaufort Gyre in summer [18]. The increasing temperature of seawater causes sea-ice to melt in the Arctic Ocean and results in a change in water mass, reflected by a decreased surface water salinity, which can be caused also by freshwater input from surrounding continents. Furthermore, the silicate concentration in the Pacific water increases during the transition across the Chukchi Sea [19]. Thus, it is not surprising that relatively high concentrations of SiO_2_ were measured in samples SW1 and SW2, despite lower nutrient concentrations in the central Arctic seawater, because the SW1 and SW2 sites were closer to the Bering Strait than the other sample sites. Samples SW1 and SW2 seem to be more heavily influenced by Pacific water than the other collected waters. Although silicate is a non-conservative tracer, it often retains a signature allowing waters from the Bering Sea to be clearly differentiated from Atlantic waters [20]. The patterns of bacterial diversity and community distribution determined in this study, specifically, that SW1 and SW2 were located slightly apart from other seawater samples in the PCoA plot (Fig. 3), support the notion of an SiO_2_ signature characterizing the Bering Sea.

In this study of the Arctic Ocean, seawaters (surface mixed layer and open melting ponds), sea-ice, and a closed melting pond were each found to harbor different bacterial communities and community compositions, with bacterial diversity in seawater samples differing from that of sea-ice and closed melting pond samples. The alpha diversity indices showed noticeable differences between seawater- and ice-related samples, which were validated by the PCoA-based community clustering. Our findings support the hypothesis that these habitats in the Arctic Ocean have their own local bacterial communities.

The difference observed between sample SW1 and the other seawater samples, as shown in the PCoA, could be explained by SW1’s much higher SiO_2_ value. Plankton, such as diatoms, take up silicate, grow, and lyse, and may thus also affect the composition of the bacterial communities, which mainly consume biopolymers such as polysaccharides released from plankton. This may also explain the differences in bacterial community composition in the Pacific water entering through the Bering Strait.

According to recent studies, the phylum *Proteobacteria* is the most dominant in the Arctic Ocean, and both the gamma and alpha subclasses occur predominantly in seawater [21]–[25]. *Gammaproteobacteria* seems to be more abundant in the surface water near coastal areas [26], [27], whereas *Alphaproteobacteria* are more abundant far from coastal areas [22], [24], [28], [29]. Moreover, in a river-influenced coastal ecosystem, the dominant bacteria in river waters are *Betaproteobacteria*, which are gradually replaced by *Alphaproteobacteria* further offshore [30]. These reports elucidate distinct bacterial community compositions in coastal and terrestrial areas, and indicating that the seawater samples and data collected in this study are unlikely to be affected by waters from coastal and terrestrial areas. For instance, in our results, the proportion of *Gammaproteobacteria* was relatively small compared to other major taxa, such as *Alphaproteobacteria* and *Flavobacteria*.

Pacific water enters the Arctic Ocean through the Bering Strait and is under the influence of fresh water in summer. We found that the oceanographic events of the Pacific sector affect the bacterial communities in the surface mixed layer water. The distribution pattern of the bacterial communities in the seawater was clearly distinguished in the abundance of *Alphaproteobacteria* and *Flavobacteria*. We recognized that temperature is not a significant influence on the assembly of the bacterial communities in the Arctic Ocean. Although most bacteria appear to be living at temperatures below their optimum growth in permanently cold polar waters, they can achieve activity rates in summer that are as high as those in temperate waters [31]. Therefore, it was assumed that the bacterial communities in the Arctic seawater might not be directly affected by temperature.

The salinity change caused by sea-ice melting may cause major bacterial fluctuations, at least in the surface water. The SAR11 clade is the most abundant and ubiquitous of the bacterial communities, but its variation differed among habitats in the Arctic Ocean. The SAR11 clade is one of the most successful groups of organisms on the planet, and could have a large impact on the cycling of carbon and other important nutrients in the oceans [32]. However, many questions remain unanswered concerning its ecology and physiology. Several studies have demonstrated that the SAR11 clade constitutes several subgroups with different preferences for environmental and biologic variables [33]–[36]. SAR11 subgroups seemed to reflect the wide salinity tolerance of a marine or brackish group [36]. This assumption is supported by the higher abundance of SAR11 group I members in seawater samples with variable salinities in this study. However, a species competition within a bacterial community or symbiotic relationships with plankton groups was not a large consideration in this study. For example, marine SAR11 require exogenous reduced sulfur originating from other plankton for growth [37]. If such accompanying taxa or specific compounds are absent in changed environments, SAR11 might be in transition. Overall, more research is needed to determine whether changes in the salinity directly affect bacterial groups, especially the SAR11 group I, or if there are other factors (not assessed in this study) affecting the bacterial communities.

The RCA clade, together with SAR11, constituted a high proportion of the marine bacteria in this study, reaching over 15% in a seawater sample (SW9; Table 3). This result confirms the prevalence of the RCA clade in the western Arctic Ocean, which was previously revealed using FISH and cloning/sequencing of 16S rRNA genes [27]. Taken together with studies that showed the abundance of the RCA clade in various Southern Ocean regions [38], our findings suggest the ecological importance of the RCA clade in both polar oceans.

The dominant *Alphaproteobacteria* in the seawater-related samples were sharply reduced in the ice-related samples. In contrast, *Flavobacteria*, which are present in lower levels in the seawater-related samples, were relatively higher in the ice-related samples. This pattern is similar to the results obtained from a summer sampling of Arctic multiyear ice [25]. However, it may not be accurate to state that the clade-specific distribution patterns between seawater- and ice-related samples depend on a salinity change because these two habitats harbor their own local communities from different assembly processes. We hypothesized that the assembly of bacterial communities in the ice-related samples is affected by 1) an elimination of the seawater-origin species with salting-out during sea-ice formation, 2) an adaptation of rare seawater species to ice conditions, or 3) an in-flow of foreign species from snow. When the surface water is frozen, highly salty and dense water is released, and sinks. We assume that the salty and dense water may sink with many species of the seawater community, leading to the overall loss in seawater-specific clades from the ice-related samples. On the other hand, the ice-specific clades were absent or rare in the seawater-related samples, which suggest that rare species of the seawater community reassembled a local community in ice-related samples. If rare species were the seed, they would be expected to be rare under certain environmental conditions and abundant when the conditions become adequate for their growth [39]. Moreover, the ice-related samples could develop their local communities from foreign bacterial assemblages, as well as the rare biospheres of seawater [28]. *Persicivirga*, *Candidatus endobugula,* and the BD7-8 marine group are respectively known as bacterial symbionts of marine algae, bryozoan, and clam [40]–[42]. The existence of these clusters in the ice-related samples might indicate the impaction of ice-associated blooms or particles from costal sediments trapped in sea-ice. *Actinobacteria* and *Bacillus* are gram-positive bacteria, and many of them are spore-formers and pigment-producers, which are characteristic of bioaerosols. These phylotypes were also discovered from early spring snow samples in the Arctic [43], suggesting that the upper layer of sea-ice is exposed to bioaerosols from snow [44]. Genus *Octadecabacter* includes gas vacuolated bacteria strains. There are two hypotheses about gas vacuolated bacteria in ice communities [45]. One possibility is that the polar gas vacuolated strains produce gas vesicles that rise up in the water column to the bottom of sea-ice so that they can reside close to the primary producers of the sea-ice bacterial community. An alternative hypothesis is that the gas vesicles allow the cells to rise to the surface of the water column during winter when sea-ice is forming so they can be present in the ice when the sea-ice bacterial community develops. We assume that the population dynamics of the ice-specific clades may be influenced by the melting process in sea-ice. During the process of ice formation, seawater is first frozen onto the bottom of the existing ice sheet in the early stage, and the ice grows thicker. In contrast, during ice melt, the uppermost layer first melts by solar radiation and then forms a melting pond on the ice sheet until it connects to seawater. In this melting process, the melted freshwater accumulates into the melting pond. Thus, the specific clades observed in the ice-related samples might remain from the sea-ice formation, snow, or other external events and persist inter-annually in sea-ice. In this study, it is difficult to explain the subtle differences in bacterial proportions within ice-related samples because only one ice core was analyzed without any physicochemical parameters. Our hypotheses for the ice-related samples need to be confirmed in further studies. Nevertheless, the ice-related samples have been an important comparison group for the seawater-related samples.

The variation of bacterial communities in the surface mixed layer water was represented by seawater-specific clades and was largely related to the sea-ice melt rather than the geographic distance. The species-sorting model provided an interpretation for this pattern. In the species-sorting process, the assembly of bacterial community can be regulated by the local environment, including selection of taxa by abiotic conditions and inter-specific competition. Therefore, if the species-sorting process is effective, different bacterial communities should occur in different habitats. Moreover, at least some of taxa in bacterial communities should resemble those in a similar habitat. Our results indicate that species-sorting operated during the re-assembly of bacterial communities from the salinity change in the surface mixed layer water. Bacterial communities of the high-silicate, high-salinity, and low-temperature Pacific water stations (SW1 and SW2) were loosely clustered within a seawater group in the PCoA, compared to the other seawater samples (from SW3 to MP3). Moreover, most of the seawater-specific clades were ubiquitous in seawater but relatively lower in the Pacific Ocean water stations. This pattern is remarkably shown by the generalist clades, ARC1 (SAR11 group I) and ARC2 (RAC clade), which were found in all samples, irrespective of habitat. It is possible that generalists were not completely sorted by the different local conditions because they had larger niche widths than the other seawater-specific clades. On the other hand, the specialist clades, ARC6 (*Polaribacter*) and ARC9 (*Balneatrix*), occurred almost exclusively in the Pacific water stations, supporting species-sorting in the Pacific sector.

There are other factors, apart from species-sorting, that can explain the assembly of communities. In particular, patch dynamics emphasizes colonization-extinction dynamics between local communities. The difference of local communities between the seawater-related and ice-related samples may be explained by patch dynamics. We did not address patch dynamics, however, because the number of samples collected from ice habitat is not enough for inferential statistics.

In this study, we used a 454-pyrosequencing method at a low sampling depth, and comparatively few sequences were analyzed for each sample. While this approach likely overlooked most of the rare taxa, the goal of this study was not to examine the full diversity in the samples, and we used 454-sequencing as a tool to assess the dramatically changing Arctic Ocean during the sea-ice melting season. We found that a change in bacterial communities occurred in the surface mixed layer water during sea-ice melt. These results were also applied to the species-sorting model to interpret the patterns. We believe that inferential statistics could be more precisely evaluated from a deeper-sampling effort, with a greater number of samples and sequence reads. For example, although sea-ice melt seems to affect population dynamics in seawater, we failed to a significant correlation between the population dynamics of SAR11 group I and the RCA clade and the salinity change, as was found for *Alphaproteobacteria*. More thorough and extensive sampling in the future will allow us to perform more detailed statistical analyses and also to develop more accurate hypotheses.

## Conclusions

Arctic seawater exhibited major changes in bacterial communities during the ice-melting season. The salinity of seawater drastically changed during the investigation period. The bacterial communities of the surface water were also affected during the ice melt and completely differed in their abundance and composition between seawater and ice samples, which represents their local communities. We applied the species-sorting model to explain population dynamics within the seawater community and found a link with salinity in the surface mixed layer of the Arctic Ocean during sea-ice melting. Although the measured environmental parameters, collected samples, and projected sequence reads were sufficient to estimate general characteristics of the Arctic seawater, we recognize that the statistical hypotheses inferred from our results can be better developed from a more accurate survey that reflects the *in situ* environment of the Arctic Ocean.

Although various types of comprehensive estimations are required to assess the dramatically changing Arctic Ocean, there are few established methods for linking microbiologic aspects with other physicochemical estimations. To monitor the Arctic sea-ice condition, satellite data have allowed for tracking the extent of sea-ice since 1978, but are limited to evaluations of environmental effects. It is important to determine environmental effects on bacterial diversity in the Arctic Ocean and whether changes in bacterial diversity affect the rates of carbon cycling and elemental cycling pathways with future consequences for the pelagic and benthic food webs [46]–[50]. Some important techniques include the benefits of synergy offered by combining data from different tools to monitor the affected bacterial diversity. In particular, abundant taxonomic groups are thought to be well-adapted to their environment and to contribute the most to biomass production [51], [52] and are easy to detect using common molecular tools.

## Supporting Information

Figure S1 **Description of the sea-ice station.** Melting ponds and ice core samples were collected from the same sea-ice sheet and isolated from each other within 10 m. (A) Open melting ponds were connected to seawater, shown as holes in the sea-ice sheet, and are represented by MP1, MP2, and MP3; their salinities were all measured to 26.0 psu. (B) The closed melting pond was isolated from seawater and is represented by MP4 (salinity: 13.0 psu). (C) The length of the sea-ice core was 103 cm and was divided into bottom (61 cm; salinity: 2.4 psu) and top (41 cm; salinity: 1.6 psu) sections.
(TIF)

Figure S2 **Distribution of nutrients in seawater.** (A) PO_4_^3−^, (B) NO_2_^−^+NO_3_^−^, (C) NH_4_^+^, and (D) SiO_2_ are represented by nine seawater samples from SW1 to SW9. The Y-axis represents deviations from an averaged value of each nutrient type.
(TIF)

Figure S3 **Phylogenetic tree construction.** Among the 26 major OTUs, a total of 22 representative sequences were analyzed; the remaining 4 were three Gram-positive bacteria and an unknown sequence. A neighbor-joining tree shows the correspondence between the representative sequences of the 22 OTUs and polar bacterial clone sequences from the ARB database. The bar indicates a Maximum Composite Likelihood distance of 0.05. Bootstrap values of 100 replicates are indicated at the node.
(TIF)

Figure S4 **Linear regression analysis for (A)** ***Alphaproteobacteria*** **and (B)** ***Flavobacteria*** **based on salinity.** Normal probability distribution, outlier values, and independence of data were described in the normal p-p plot and the scatter plot of standardized residual and auto-regression (Durbin-Watson), respectively.
(TIF)

Table S1 **GPS information and physicochemical parameter values in each sample.** Samples of MP1, MP2, and MP3 were collected from melting ponds that opened to the surface water, and MP4 was collected from a closed melting pond (described in the Fig. S1A and B). Samples of IB and IT, respectively, indicate the lower and upper part of the ice core (Fig. S1C). Theses samples were all collected from same sea-ice sheet, and their physicochemical parameters other than salinity were not estimated (ND). Column headings: Lon. = longitude, Lat. = latitude, Sal. = measured salinity, Temp. = measured temperature, Fluor. = concentration of fluorescing molecules such as chlorophyll a and colored dissolved organic matter, PO_4_^3−^ = concentration of PO_4_^3−^, NO_2_^−^+NO_3_^−^ = concentration of NO_2_^−^+NO_3_^−^, NH_4_^+^ = concentration of NH_4_^+^, SiO_2_ = concentration of SiO_2_.
(DOCX)

Table S2 **Bacterial community in class level.**
(ZIP)
