# Supplementary figures and images for: Bacterial Communities of Surface Mixed Layer in the Pacific Sector of the Western Arctic Ocean during Sea-Ice Melting
Source: PLoS One. 2014 Jan 31;9(1):e86887. doi: 10.1371/journal.pone.0086887 (PMC3908934; doi:10.1371/journal.pone.0086887)

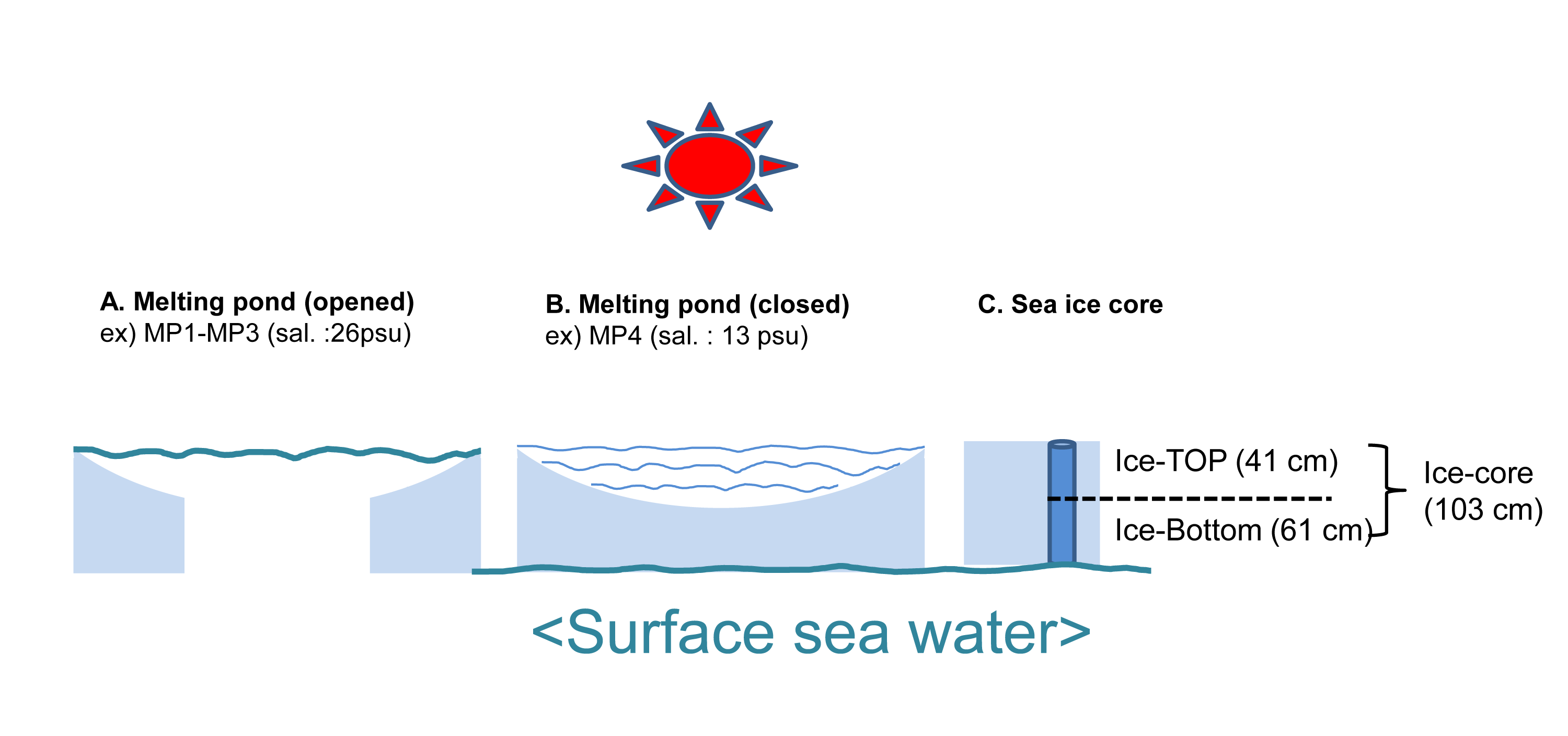

Supplement: Figure S1 — Description of the sea-ice station. Melting ponds and ice core samples were collected from the same sea-ice sheet and isolated from each other within 10 m. (A) Open melting ponds were connected to seawater, shown as holes in the sea-ice sheet, and are represented by MP1, MP2, and MP3; their salinities were all measured to 26.0 psu. (B) The closed melting pond was isolated from seawater and is represented by MP4 (salinity: 13.0 psu). (C) The length of the sea-ice core was 103 cm and was divided into bottom (61 cm; salinity: 2.4 psu) and top (41 cm; salinity: 1.6 psu) sections. (TIF) [file pone.0086887.s001.tif]

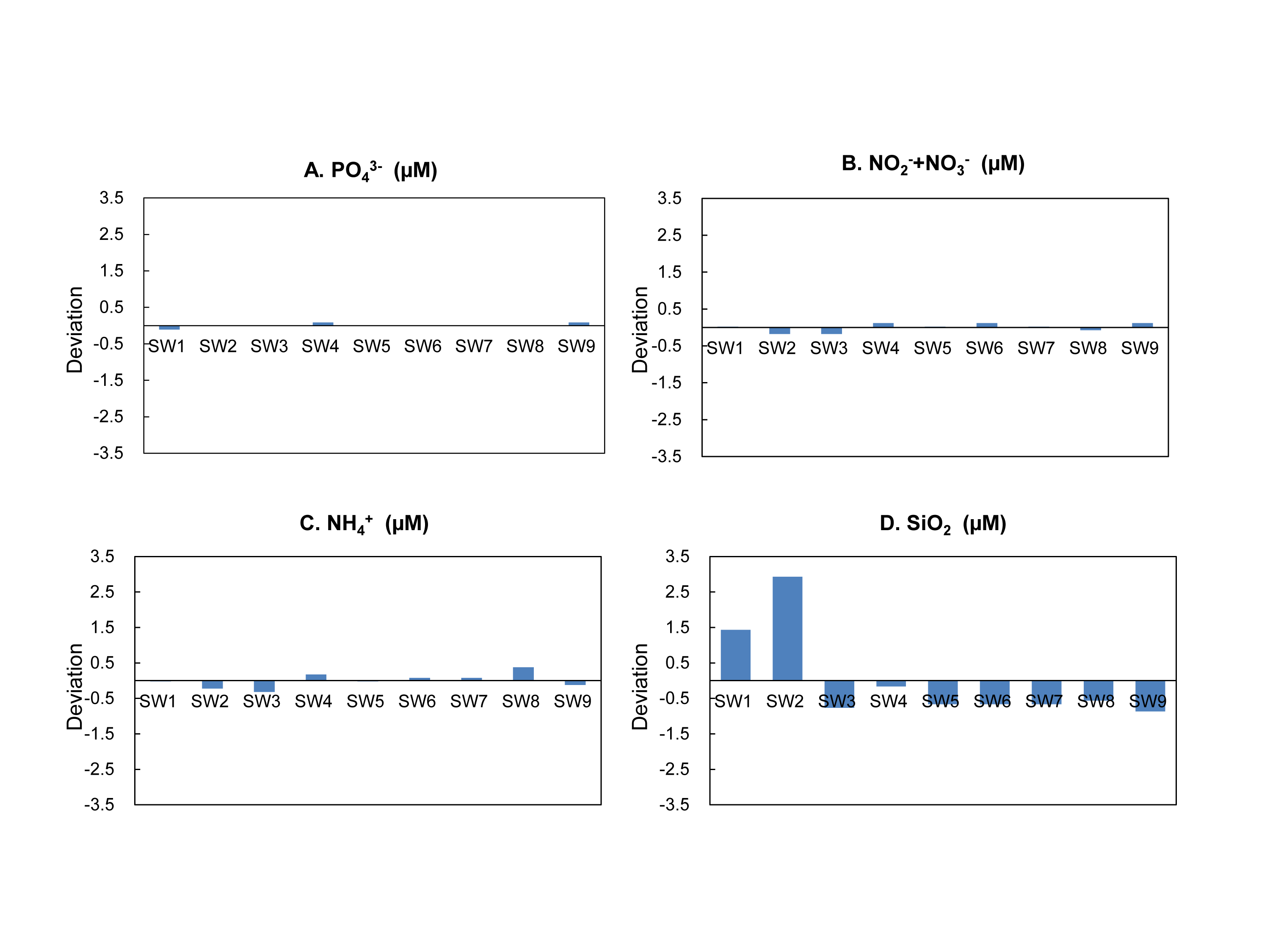

Supplement: Figure S2 — Distribution of nutrients in seawater. (A) PO4 3−, (B) NO2 −+NO3 −, (C) NH4 +, and (D) SiO2 are represented by nine seawater samples from SW1 to SW9. The Y-axis represents deviations from an averaged value of each nutrient type. (TIF) [file pone.0086887.s002.tif]

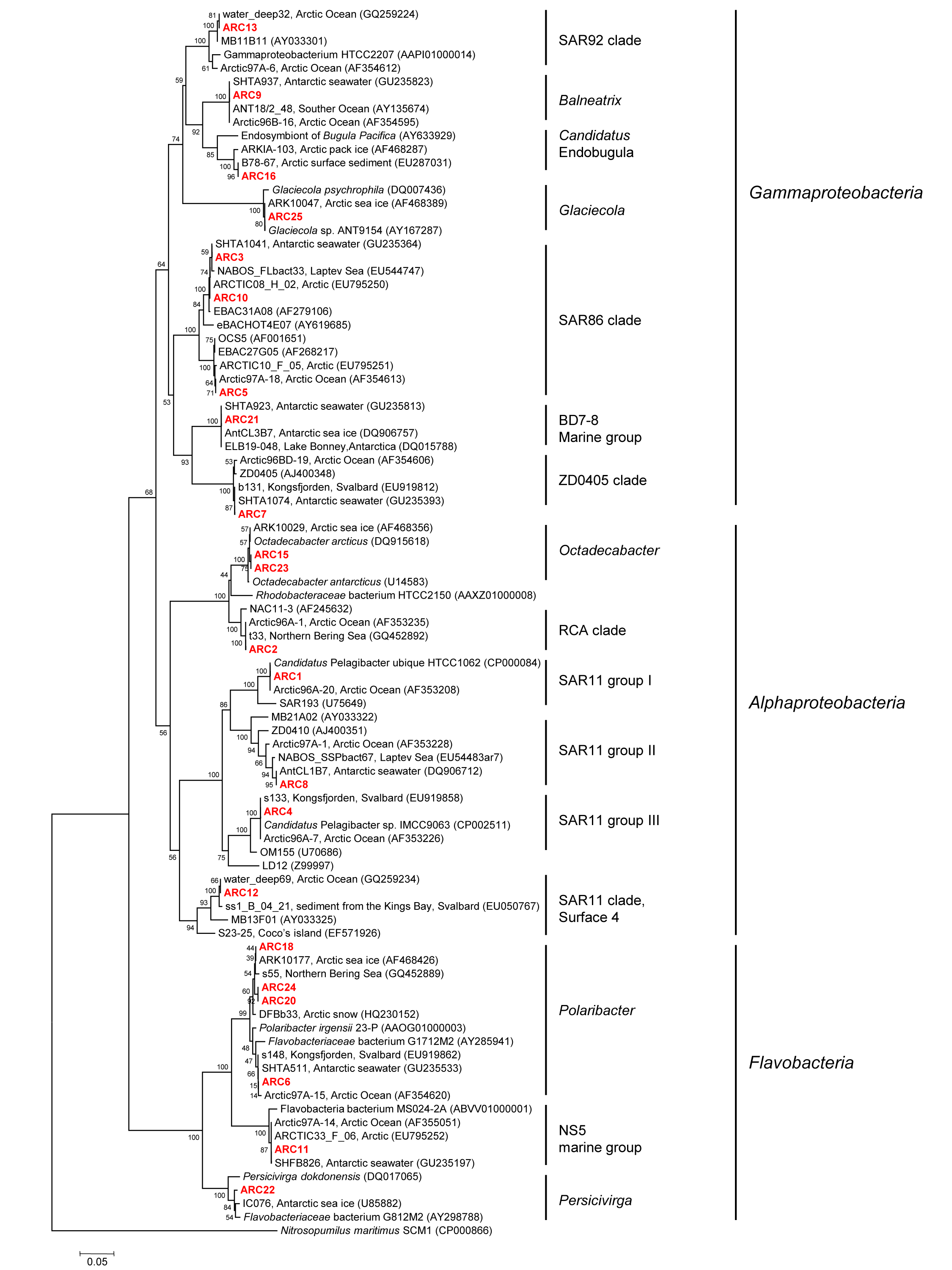

Supplement: Figure S3 — Phylogenetic tree construction. Among the 26 major OTUs, a total of 22 representative sequences were analyzed; the remaining 4 were three Gram-positive bacteria and an unknown sequence. A neighbor-joining tree shows the correspondence between the representative sequences of the 22 OTUs and polar bacterial clone sequences from the ARB database. The bar indicates a Maximum Composite Likelihood distance of 0.05. Bootstrap values of 100 replicates are indicated at the node. (TIF) [file pone.0086887.s003.tif]

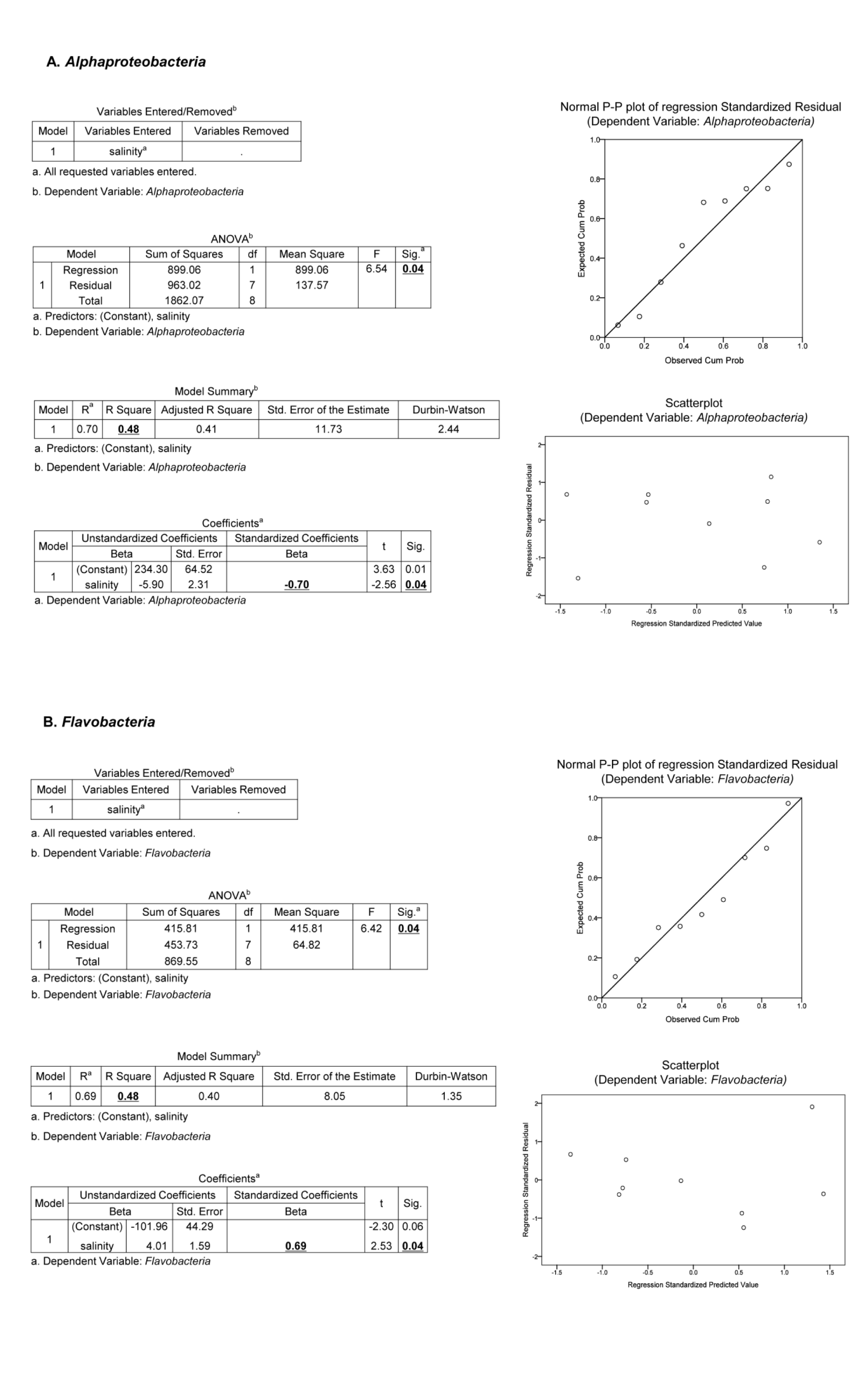

Supplement: Figure S4 — Linear regression analysis for (A) Alphaproteobacteria and (B) Flavobacteria based on salinity. Normal probability distribution, outlier values, and independence of data were described in the normal p-p plot and the scatter plot of standardized residual and auto-regression (Durbin-Watson), respectively. (TIF) [file pone.0086887.s004.tif]
