# Supplementary material for: Bacterial Communities of Surface Mixed Layer in the Pacific Sector of the Western Arctic Ocean during Sea-Ice Melting
Source: PLoS One. 2014 Jan 31;9(1):e86887. doi: 10.1371/journal.pone.0086887 (PMC3908934; doi:10.1371/journal.pone.0086887)
Supplement: Table S1 — GPS information and physicochemical parameter values in each sample. Samples of MP1, MP2, and MP3 were collected from melting ponds that opened to the surface water, and MP4 was collected from a closed melting pond (described in the Fig. S1A and B). Samples of IB and IT, respectively, indicate the lower and upper part of the ice core (Fig. S1C). Theses samples were all collected from same sea-ice sheet, and their physicochemical parameters other than salinity were not estimated (ND). Column headings: Lon. = longitude, Lat. = latitude, Sal. = measured salinity, Temp. = measured temperature, Fluor. = concentration of fluorescing molecules such as chlorophyll a and colored dissolved organic matter, PO4 3− = concentration of PO4 3−, NO2 −+NO3 − = concentration of NO2 −+NO3 −, NH4 + = concentration of NH4 +, SiO2 = concentration of SiO2. (DOCX) [file pone.0086887.s005.docx]

| **Sample** | **GPS** | | **Physiochemical parameters** | | | | | | |
| --- | --- | --- | --- | --- | --- | --- | --- | --- | --- |
|  | **Lat. (N)** | **Lon. (W)** | **Sal. (psu)** | **Temp. (°C)** | **Fluor. (mg/m^3^)** | **PO_4_^3-^ (µM)** | **NO_2_^-^+NO_3_^-^ (µM)** | **NH_4_^+^ (µM)** | **SiO_2_ (µM)** |
| SW1 | 73° 07.68' | 168° 56.07' | 30.2 | -1.5 | 0.5 | 0.5 | 0.3 | 0.5 | 4.8 |
| SW2 | 73° 01.50' | 168° 30.92' | 30.5 | -1.5 | 0.7 | 0.6 | 0.1 | 0.3 | 6.3 |
| SW3 | 73° 30.95' | 166° 58.87' | 28.9 | -1.4 | 0.6 | 0.6 | 0.1 | 0.2 | 2.6 |
| SW4 | 73° 44.91' | 167° 01.99' | 28.8 | -1.4 | 0.6 | 0.7 | 0.4 | 0.7 | 3.2 |
| SW5 | 74° 59.97' | 159° 59.98' | 26.5 | -1.2 | 0.6 | 0.6 | 0.3 | 0.5 | 2.7 |
| SW6 | 75° 00.03' | 159° 01.95' | 26.6 | -0.6 | 0.7 | 0.6 | 0.4 | 0.6 | 2.7 |
| SW7 | 74° 57.72' | 156° 14.92' | 26.4 | -1.1 | 0.7 | 0.6 | 0.3 | 0.6 | 2.7 |
| SW8 | 75° 58.90' | 156° 26.66' | 25.5 | -1.1 | 0.6 | 0.6 | 0.2 | 0.9 | 2.8 |
| SW9 | 78° 00.56' | 160° 02.02' | 27.6 | -1.4 | 0.5 | 0.7 | 0.4 | 0.4 | 2.5 |
| MP1 | 77° 02.66' | 159° 46.99' | 26.0 | ND | ND | ND | ND | ND | ND |
| MP2 | " | " | " | " | " | " | " | " | " |
| MP3 | " | " | " | " | " | " | " | " | " |
| MP4 | " | " | 13.0 | " | " | " | " | " | " |
| IB | " | " | 2.4 | " | " | " | " | " | " |
| IT | " | " | 1.6 | " | " | " | " | " | " |

Table S1. GPS information and physicochemical parameter values in each sample**.**

Samples of MP1, MP2, and MP3 were collected from melting ponds that opened to the surface water, and MP4 was collected from a closed melting pond (described in the Fig. S1a and b) Samples of IB and IT, respectively, indicate the lower and upper part of the ice core (Fig. S1c). Theses samples were all collected from same sea-ice sheet, and their physicochemical parameters other than salinity were not estimated (ND).

Column headings: Lon. = longitude, Lat. = latitude, Sal. = measured salinity, Temp. = measured temperature, Fluor. = concentration of fluorescing molecules such as chlorophyll a and colored dissolved organic matter, PO43- = concentration of PO43-, NO2-+NO3- = concentration of NO2-+NO3-, NH4+ = concentration of NH4+, SiO2 = concentration of SiO2,
