# Supplementary material for: Bacterial Communities of Surface Mixed Layer in the Pacific Sector of the Western Arctic Ocean during Sea-Ice Melting
Source: PLoS One. 2014 Jan 31;9(1):e86887. doi: 10.1371/journal.pone.0086887 (PMC3908934; doi:10.1371/journal.pone.0086887)
Supplement: Table S2 — Bacterial community in class level. (ZIP) [file pone.0086887.s006.zip › Table S2.docx]

| **Class** | **SW1** | **SW2** | **SW3** | **SW4** | **SW5** | **SW6** | **SW7** | **SW8** | **SW9** | **MP1** | **MP2** | **MP3** | **MP4** | **IB** | **IT** | **Total** |
| --- | --- | --- | --- | --- | --- | --- | --- | --- | --- | --- | --- | --- | --- | --- | --- | --- |
| **Alphaproteobacteria** | 37.77 | 62.50 | 69.35 | 71.95 | 83.69 | 62.82 | 91.78 | 77.11 | 70.03 | 80.19 | 83.36 | 84.66 | 7.59 | 35.05 | 35.15 | 62.82 |
| **Flavobacteria** | 34.77 | 17.37 | 3.94 | 6.84 | 2.70 | 8.90 | 1.03 | 5.64 | 8.83 | 5.98 | 6.10 | 5.07 | 76.37 | 21.10 | 27.62 | 15.76 |
| **Gammaproteobacteria** | 25.28 | 16.16 | 24.88 | 19.24 | 12.68 | 21.50 | 6.40 | 15.30 | 17.03 | 12.16 | 8.43 | 8.99 | 11.21 | 20.86 | 11.34 | 15.62 |
| **Bacilli** | 0.00 | 0.00 | 0.00 | 0.00 | 0.00 | 0.06 | 0.00 | 0.00 | 0.00 | 0.00 | 0.00 | 0.00 | 0.00 | 8.99 | 12.84 | 1.71 |
| **Chloroplast** | 0.02 | 0.00 | 0.00 | 0.04 | 0.00 | 0.00 | 0.00 | 0.05 | 0.57 | 0.35 | 0.49 | 0.25 | 3.13 | 4.52 | 5.22 | 1.04 |
| **Actinobacteria (class)** | 0.24 | 0.17 | 0.82 | 0.87 | 0.00 | 1.23 | 0.00 | 0.38 | 0.50 | 0.45 | 0.41 | 0.17 | 0.25 | 3.24 | 4.91 | 1.02 |
| **Betaproteobacteria** | 0.33 | 0.17 | 0.09 | 0.32 | 0.70 | 3.58 | 0.74 | 1.07 | 2.36 | 0.52 | 0.71 | 0.44 | 0.08 | 0.93 | 0.42 | 0.77 |
| **Sphingobacteria** | 1.54 | 3.62 | 0.39 | 0.59 | 0.23 | 1.79 | 0.05 | 0.41 | 0.42 | 0.23 | 0.36 | 0.33 | 0.11 | 1.72 | 0.23 | 0.69 |
| **Nostocophycideae** | 0.00 | 0.00 | 0.00 | 0.00 | 0.00 | 0.00 | 0.00 | 0.00 | 0.00 | 0.00 | 0.03 | 0.00 | 0.06 | 2.45 | 1.04 | 0.24 |
| **Epsilonproteobacteria** | 0.00 | 0.00 | 0.00 | 0.00 | 0.00 | 0.00 | 0.00 | 0.00 | 0.00 | 0.09 | 0.03 | 0.00 | 1.19 | 0.18 | 0.28 | 0.11 |
| **A712011** | 0.00 | 0.00 | 0.00 | 0.00 | 0.00 | 0.00 | 0.00 | 0.00 | 0.00 | 0.00 | 0.00 | 0.00 | 0.00 | 0.79 | 0.21 | 0.06 |
| **Synechococcophycideae** | 0.00 | 0.00 | 0.00 | 0.00 | 0.00 | 0.00 | 0.00 | 0.00 | 0.00 | 0.00 | 0.00 | 0.00 | 0.00 | 0.06 | 0.49 | 0.05 |
| **Deltaproteobacteria** | 0.00 | 0.00 | 0.00 | 0.06 | 0.00 | 0.00 | 0.00 | 0.00 | 0.21 | 0.00 | 0.00 | 0.00 | 0.00 | 0.00 | 0.02 | 0.02 |
| **Acidobacteria (class)** | 0.00 | 0.00 | 0.17 | 0.00 | 0.00 | 0.00 | 0.00 | 0.00 | 0.00 | 0.00 | 0.00 | 0.00 | 0.00 | 0.00 | 0.07 | 0.01 |
| **TM7-3** | 0.00 | 0.00 | 0.00 | 0.00 | 0.00 | 0.00 | 0.00 | 0.02 | 0.02 | 0.00 | 0.00 | 0.00 | 0.00 | 0.06 | 0.00 | 0.01 |
| **Opitutae** | 0.05 | 0.00 | 0.00 | 0.00 | 0.00 | 0.00 | 0.00 | 0.00 | 0.00 | 0.00 | 0.03 | 0.02 | 0.00 | 0.00 | 0.00 | 0.01 |
| **Clostridia** | 0.00 | 0.00 | 0.00 | 0.04 | 0.00 | 0.00 | 0.00 | 0.00 | 0.00 | 0.00 | 0.00 | 0.00 | 0.00 | 0.00 | 0.03 | 0.01 |
| **AB16** | 0.02 | 0.00 | 0.00 | 0.01 | 0.00 | 0.00 | 0.00 | 0.00 | 0.02 | 0.00 | 0.00 | 0.00 | 0.00 | 0.00 | 0.00 | 0.00 |
| **Oscillatoriophycideae** | 0.00 | 0.00 | 0.00 | 0.00 | 0.00 | 0.00 | 0.00 | 0.00 | 0.00 | 0.00 | 0.00 | 0.04 | 0.00 | 0.03 | 0.00 | 0.00 |
| **Solibacteres** | 0.00 | 0.00 | 0.13 | 0.00 | 0.00 | 0.00 | 0.00 | 0.00 | 0.00 | 0.00 | 0.00 | 0.00 | 0.00 | 0.00 | 0.00 | 0.00 |
| **Unknown** | 0.00 | 0.00 | 0.00 | 0.00 | 0.00 | 0.00 | 0.00 | 0.00 | 0.00 | 0.00 | 0.00 | 0.00 | 0.00 | 0.03 | 0.03 | 0.00 |
| **Deinococci** | 0.00 | 0.00 | 0.00 | 0.00 | 0.00 | 0.00 | 0.00 | 0.01 | 0.00 | 0.02 | 0.00 | 0.00 | 0.00 | 0.00 | 0.00 | 0.00 |
| **Chloroflexi-4** | 0.00 | 0.00 | 0.00 | 0.00 | 0.00 | 0.11 | 0.00 | 0.00 | 0.00 | 0.00 | 0.00 | 0.00 | 0.00 | 0.00 | 0.00 | 0.00 |
| **SOGA31** | 0.00 | 0.00 | 0.04 | 0.00 | 0.00 | 0.00 | 0.00 | 0.00 | 0.00 | 0.00 | 0.00 | 0.00 | 0.00 | 0.00 | 0.02 | 0.00 |
| **Chloracidobacteria** | 0.00 | 0.00 | 0.00 | 0.00 | 0.00 | 0.00 | 0.00 | 0.00 | 0.00 | 0.00 | 0.00 | 0.00 | 0.00 | 0.00 | 0.03 | 0.00 |
| **Planctomycea** | 0.00 | 0.00 | 0.00 | 0.00 | 0.00 | 0.00 | 0.00 | 0.00 | 0.00 | 0.00 | 0.00 | 0.00 | 0.00 | 0.00 | 0.03 | 0.00 |
| **Verrucomicrobiae** | 0.00 | 0.00 | 0.00 | 0.00 | 0.00 | 0.00 | 0.00 | 0.01 | 0.00 | 0.00 | 0.00 | 0.00 | 0.00 | 0.00 | 0.00 | 0.00 |
| **BD4-9** | 0.00 | 0.00 | 0.00 | 0.01 | 0.00 | 0.00 | 0.00 | 0.00 | 0.00 | 0.00 | 0.00 | 0.00 | 0.00 | 0.00 | 0.00 | 0.00 |
| **Phycisphaerae** | 0.00 | 0.00 | 0.00 | 0.00 | 0.00 | 0.00 | 0.00 | 0.00 | 0.00 | 0.00 | 0.00 | 0.02 | 0.00 | 0.00 | 0.00 | 0.00 |
| **SJA-176** | 0.00 | 0.00 | 0.04 | 0.00 | 0.00 | 0.00 | 0.00 | 0.00 | 0.00 | 0.00 | 0.00 | 0.00 | 0.00 | 0.00 | 0.00 | 0.00 |
| **Unclassified** | 0.00 | 0.00 | 0.04 | 0.00 | 0.00 | 0.00 | 0.00 | 0.00 | 0.00 | 0.00 | 0.00 | 0.00 | 0.00 | 0.00 | 0.00 | 0.00 |
| **Elusimicrobia (class)** | 0.00 | 0.00 | 0.04 | 0.00 | 0.00 | 0.00 | 0.00 | 0.00 | 0.00 | 0.00 | 0.00 | 0.00 | 0.00 | 0.00 | 0.00 | 0.00 |
| **Armatimonadia** | 0.00 | 0.00 | 0.04 | 0.00 | 0.00 | 0.00 | 0.00 | 0.00 | 0.00 | 0.00 | 0.00 | 0.00 | 0.00 | 0.00 | 0.00 | 0.00 |
| **SJA-28** | 0.00 | 0.00 | 0.00 | 0.00 | 0.00 | 0.00 | 0.00 | 0.00 | 0.00 | 0.00 | 0.03 | 0.00 | 0.00 | 0.00 | 0.00 | 0.00 |
| **Bacteroidia** | 0.00 | 0.00 | 0.00 | 0.00 | 0.00 | 0.00 | 0.00 | 0.00 | 0.00 | 0.00 | 0.03 | 0.00 | 0.00 | 0.00 | 0.00 | 0.00 |
| **SJA-4** | 0.00 | 0.00 | 0.00 | 0.00 | 0.00 | 0.00 | 0.00 | 0.00 | 0.00 | 0.00 | 0.00 | 0.00 | 0.00 | 0.00 | 0.02 | 0.00 |
